# Transferrin Non-Viral Gene Therapy for Treatment of Retinal Degeneration

**DOI:** 10.3390/pharmaceutics12090836

**Published:** 2020-09-01

**Authors:** Karine Bigot, Pauline Gondouin, Romain Bénard, Pierrick Montagne, Jenny Youale, Marie Piazza, Emilie Picard, Thierry Bordet, Francine Behar-Cohen

**Affiliations:** 1 Eyevensys, Biopark, 11 rue Watt, 75013 Paris, France; karine.bigot@eyevensys.com (K.B.); pauline.gondouin@eyevensys.com (P.G.); romain.benard@eyevensys.com (R.B.); pierrick.montagne@eyevensys.com (P.M.); jenny.youale@eyevensys.com (J.Y.); marie.piazza@eyevensys.com (M.P.); 2 Centre de Recherche des Cordeliers, INSERM, Sorbonne Université, USPC, Université Paris Descartes, Team 17, 75006 Paris, France; emilie.picard@crc.jussieu.fr; 3 Ophtalmopole, Cochin Hospital, AP-HP, Assistance Publique Hôpitaux de Paris, 24 rue du Faubourg Saint-Jacques, 75014 Paris, France

**Keywords:** iron, retinal degeneration, age-related macular degeneration, retinitis pigmentosa, transferrin, gene therapy, plasmid electrotransfection

## Abstract

Dysregulation of iron metabolism is observed in animal models of retinitis pigmentosa (RP) and in patients with age-related macular degeneration (AMD), possibly contributing to oxidative damage of the retina. Transferrin (TF), an endogenous iron chelator, was proposed as a therapeutic candidate. Here, the efficacy of TF non-viral gene therapy based on the electrotransfection of pEYS611, a plasmid encoding human TF, into the ciliary muscle was evaluated in several rat models of retinal degeneration. pEYS611 administration allowed for the sustained intraocular production of TF for at least 3 and 6 months in rats and rabbits, respectively. In the photo-oxidative damage model, pEYS611 protected both retinal structure and function more efficiently than carnosic acid, a natural antioxidant, reduced microglial infiltration in the outer retina and preserved the integrity of the outer retinal barrier. pEYS611 also protected photoreceptors from N-methyl-N-nitrosourea-induced apoptosis. Finally, pEYS611 delayed structural and functional degeneration in the RCS rat model of RP while malondialdehyde (MDA) ocular content, a biomarker of oxidative stress, was decreased. The neuroprotective benefits of TF non-viral gene delivery in retinal degenerative disease models further validates iron overload as a therapeutic target and supports the continued development of pEY611 for treatment of RP and dry AMD.

## 1. Introduction

Iron is essential for retinal metabolism and visual cycle, but excessive ferrous iron (Fe^2+^) can generate reactive oxygen species (ROS) and consecutive oxidative damage to the cellular environment (reviewed in [1,2]). Iron particles cause oxidative damage to rat photoreceptors, with greater damage to cones than rods [3] and iron-induced photoreceptor cell death was associated with lipid peroxidation [4], activation of the nucleotide-binding oligomerization domain (NOD)-like receptor family pyrin domain-containing 3 (NLRP3) inflammasome signaling pathway, and induction of necrotic and apoptotic markers [3,5,6]. In addition, the phagocytosis of Fe^2+^-oxidized photoreceptor outer segments further damage the retinal pigment epithelium (RPE) [7], markedly decreasing phagocytosis activity and lysosomal function [8], and possibly inducing RPE de-differentiation [9]. To tightly control the intracellular iron level, several proteins regulate iron import, storage, and export. Transferrin (TF) binds ferric iron (Fe^3+^) with a very high affinity (association constant is 10^20^ M^−1^ at pH 7.4) and is then internalized by transferrin receptors (TFRs), allowing iron to enter into the cell. Inside cells, iron dissociates from TF and enters in the labile iron pool while TF is recycled at the cell membrane. Intracellular iron is supplied to cytosolic proteins for storage (ferritins), taken up by the mitochondria or released from the cell by ferroportin, a membrane transporter requiring ferroxidases, such as ceruloplasmin or hephaestin, to convert the ferrous Fe^2+^ to ferric Fe^3+^ form. In addition, iron-sensitive intracellular proteins, such as iron-regulatory proteins (IRP1 and IRP2), hepcidin or hypoxia-inducing factor (HIF), serve as check points to control the intracellular iron concentration (recently reviewed in [2]).

Iron-mediated retinal cell death has been shown to occur in various models of retinal degeneration and suspected to occur in age-related macular degeneration (AMD) and retinitis pigmentosa (RP). Indeed, iron deposits (including labile iron) were observed in postmortem AMD macula at the RPE–choroid interface within metal-storing melanosomes, in drusens, in the central layer of the calcified Bruch’s membrane but also in the neurosensory retina [10,11,12]. The level of iron in the aqueous humor of dry AMD patients undergoing cataract surgery was two-fold higher than in age-matched controls [13] and the expression of proteins involved in iron homeostasis, such as ferritin and ferroportin, was increased in the macula of AMD patients with geographic atrophy [11]. Similarly, increased expression of TF was shown in the retinas of AMD patients relative to those of healthy control patients, which suggests that iron regulation is impaired [14]. Finally, several polymorphisms in iron homeostasis genes have been identified as risk factors for AMD, such as the TF receptors (TFR1 and TFR2) genes [15], IRP1 and IRP2 genes [16], and the heme oxygenase-1 and -2 (HMOX1/2) genes [17]. We and others have characterized the role of iron homeostasis dysregulation and associated oxidative injury in the pathogenesis of RP. In the Royal College of Surgeons (RCS) rat [18] and in the rd10 mouse [19,20], two models of RP, degeneration of the retina was associated with altered iron homeostasis and its progression correlated with iron overload. Furthermore, mutation in the feline leukemia virus subgroup C cellular receptor 1 (FLVCR1), a gene encoding a heme-transporter protein involved in iron homeostasis, is also causing syndromic autosomal-recessive RP [21].

Whether loss of iron homeostasis is a cause or a consequence of various retinal diseases remains unknown, but it is undeniable that an excess of free iron is pathogenic, and that its neutralization with chemical chelators protects the retina not only in rodent models with impaired mechanisms of iron homeostasis [22,23] but also in models of inherited retinal dystrophies [24,25,26]. Chemical iron chelators failed so far to reach clinical stage because of limited ocular bioavailability and/or ocular side-effects (cataract, retinopathy optic neuritis) probably due to chelation of intracellular iron store necessary for retina cells’ function [2,27]. Conversely, the natural iron transporter TF was proven to be safe in the eye when overexpressed in transgenic mouse or when delivered intraperitoneally or intravitreally in the rat even after repeated administration of high doses (up to 240 µg per eye) [19,28,29,30]. Thus, we proposed to use TF as a therapeutic candidate for safe iron chelation in ocular diseases. Although vitreous humor contains a high amount of TF in physiologic conditions both as the free form (apo-TF) and the iron-bound form (holo-TF) [31], the expression of TF and of its receptor is upregulated in retinal degeneration or inflammation [14,20,29] suggesting that TF levels could be a limiting factor in disease conditions. We recently reported that both the TF saturation rate and levels of free iron were increased in the vitreous and in the subretinal fluid of patients with retinal detachment, together correlated with reduced postoperative visual recovery [6], identifying TF as a good candidate to buffer free iron in the eye. When injected into the vitreous, TF distributes throughout the neural retina and the RPE via its receptors and is rapidly eliminated from the retina by a transretinal elimination route [30], suggesting that local delivery of the protein itself would require repeated administration in the context of chronic retinal diseases. Importantly, TF was highly potent to preserve photoreceptors in animal models of retinal degeneration induced by iron overload, light exposure, inherited genetic mutations, or retinal detachment [6,19,30]. In these models, TF restored iron homeostasis, reduced oxidative stress, inflammation, and cell death, preserving photoreceptor cells and visual function [6,30].

In the present study, we evaluated a non-viral gene therapy strategy that combines a plasmid coding for human TF (pEYS611), and an electrotransfection system to deliver DNA plasmids into the ciliary muscle, in order to sustainably produce apo-TF into the eye. Such a technology was previously shown to efficiently allow the production of a variety of secreted proteins, including trophic factors, anti-TNF, or anti-VEGF [32,33,34,35,36]. We investigated the therapeutic potential of pEYS611 in several rat models of induced retinal degeneration, including the RCS rat model of RP and the photo-oxidative damage model, evaluating both retinal histology and function. These findings further validate iron overload as a therapeutic target for the treatment of retinal degeneration and pEYS611 as a valuable product to neutralize ocular iron excess.

## 2. Materials and Methods

### 2.1. pEYS611 Plasmid Construct

pEYS611 is a 5201-base pair plasmid DNA coding for the human transferrin (TF). The codon-optimized cDNA sequence of TF was de novo synthesized by GeneArt (ThermoFisher Scientific, Waltham, MA, USA), subcloned into the NTC8685 plasmid backbone (Nature Technology Corporation, Lincoln, NE, USA) [37] by enzymatic restriction and verified by sequencing. Transgene expression was placed under the control of an optimized chimeric CMV-HTLV-1R ubiquitous promoter and the ß-globin intron. pEYS611 plasmid was amplified through an antibiotic-free selection procedure in modified *E. coli* cells [38] and formulated in sterile Tris-EDTA buffered saline at a final concentration of 5 mg/mL.

### 2.2. Animals

Male Wistar and Sprague Dawley rats were obtained from Janvier Labs (Le Genest-Saint-Isle, France) to conduct the light-induced damage and the N-methyl-N-nitrosourea (MNU)-induced retinal degeneration models, respectively, as previously reported (see below). New Zealand rabbits were provided by CEGAV (Saint Mars d’Egrenne, France). Pigmented dystrophic RCS and pigmented control non-dystrophic RDY rats were a gift from Dr Emeline Nandrot (Institut de la Vision, Paris, France).

Rats were group-housed (maximum 3 rats per cage) and rabbits were individually housed. Each cage contained an environmental enrichment. Animals were fed ad libitum with a diet for maintenance; they were maintained in a temperature-controlled room at 21–23 °C with the light-environment consisting of 12- or 18-h light per day for rabbits and rats, respectively.

Animals were acclimated for at least one week in the animal facility prior any experiments. All experimental methods and protocols were carried out according to standard operating procedures with approval by the Institutional Ethic Committees of Sorbonne university and National veterinary school of Alfort and French Ministry of Higher Education, Research and Innovation (certificates APAFIS#7001-2015100815363248, APAFIS#11019-2017082415542346, APAFIS#7411-2016102710489557 and APAFIS#15364-20180531109399950). In addition, experiments complied with the ARVO statement for animal experimentation and followed European guidelines for animal welfare.

### 2.3. Plasmid Electrotransfection Procedure in Rat and Rabbit Ciliary Muscle

Animals were anesthetized by intramuscular injection of a mixture of ketamine (8% Imalgen 1000, Merial, France) and xylazine (2% Rompun, Bayer AG, Leverkusen, Germany). Electrotransfection of pEYS611 in the rat ciliary muscle was performed as previously described [34]. Briefly, following protrusion of the eye with an operating field, injection of the plasmid solution (10 µL) was performed transclerally using a 30G needle parallel to the limbus. An iridium/platinum wire electrode (cathode, Good Fellow, Lille, France) was then inserted into the transscleral tunnel, and a semi-annular stainless-steel sheet electrode (anode, Good Fellow, Lille, France) was placed on the sclera, facing the wire electrode. The eye was humidified by instillation of an additional drop of 0.9% NaCl solution to facilitate conductance. Square-wave electrical pulses (200 V/cm, 5 Hz, 10 ms, 8 pulses) were generated with Eyevensys proprietary Pulse Generator.

**Figure 1:**
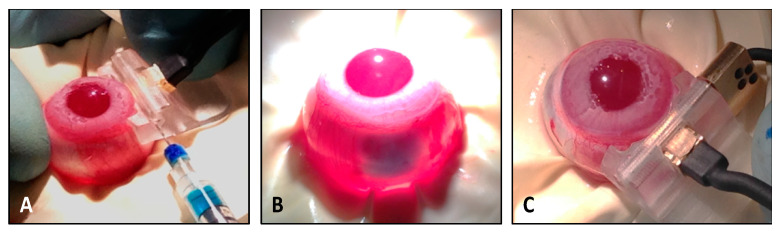
Ciliary muscle electrotransfection procedure in rabbit. (**A**) Plasmid injection in the rabbit ciliary muscle was performed using a dedicated ocular device placed on the nasal scleral surface. (**B**) Tracer dye (blue) was used for the demonstration purpose only and was not used during administration of pEYS611. (**C**) Following adjusted placement of the ocular device so that its superior part fits with the limbus, the comb electrode was inserted into ocular tissues until its stop position.

Plasmid electrotransfection in rabbits was performed using a dedicated ocular device similar to a device previously described for human application [35]. When placed on the nasal scleral surface and adjusted with the limbus, such a device allows for precise and consistent guiding of the plasmid injection into the posterior longitudinal ciliary muscle fibers using a 29G-needle (30 µL). A tracer dye insuring proper ciliary muscle injection was used for training of operators and demonstration purpose as described in Figure 1. Following instillation of two to three drops of physiological solution (0.9% NaCl) on the eye, a square-wave electrical pulse sequence was delivered in between the surface electrode and the needle electrodes inserted in the anterior vitreous just beneath the ciliary muscle. Electric pulses (high voltage = 500 V/cm, 1 Hz, 100 µs, 1 pulse duration, 10 s break; low voltage = 200 V/cm, 1 Hz, 100 ms, 4 pulses) were delivered with an Eyevensys Pulse Generator. All animals received post-operative ophthalmic gel of carbomer onto the cornea (Lacrigel, Laboratoire Europhta, Monaco).

### 2.4. Quantification of Human Transferrin (TF) in Ocular Fluids and Tissues

Quantification of TF in ocular fluids and in retinal tissues was performed using a specific enzyme-linked immunosorbent assay (ELISA). Briefly, goat anti-human TF polyclonal antibodies (Bio-Rad, Hercules, CA, USA) were coated on a 96-well high-binding affinity plate. Captured human TF was detected using a biotinylated goat polyclonal antibody raised against the human TF (Bio-Rad, Hercules, CA, USA) and revealed with streptavidin-horse radish peroxidase (HRP, Sigma-Aldrich, St. Louis, MI, USA) enzyme and tetramethylbenzidine (TMB) substrate (Sigma-Aldrich, St. Louis, MI, USA). The 450-nm absorbance was measured using the Infinite F200 plate reader (Tecan, Männedorf, Switzerland). Recombinant human TF (Bio-Rad, Hercules, CA, USA) was used as a standard. The lower limit of quantification (LLoQ) in rat ocular fluids (aqueous humor and vitreous pooled together) and in rabbit aqueous humor, vitreous and retina/RPE/choroid complex was defined as 195.3 and 39 pg/mL, respectively. Concentrations of human TF were calculated using a four-parameter logistic curve fit (4-PL, MyAssays software) from the standard curve. Values were expressed as mean ± standard error of the mean (sem) in ng/mL of fluids or ng/g of tissues.

### 2.5. Light-Induced Damage Rat Model

Male Wistar rats (4-week-old at arrival) were maintained for 3 weeks prior to light exposure in a dimly lit environment (maximum 200 lux) by placing the ventilated cages at the bottom of the rack to reduce variation in the photoreceptor sensibility between animals, as previously described [30]. Dark adaptation was performed during at least 15 h before light exposure. Animals were then placed in individual transparent cages with access to food and water (Hydrogel, Janvier Labs, Saint Berthevin, France), and retina light-induced damage (LID) was induced by exposition under a cold white LED of 6500 lux mounted on a lamp board (slightly raised to allow airflow) during 24 h. After light exposure, animals returned to normal cyclic light conditions as described above. Control animals underwent dark adaptation but then returned to rearing cyclic light conditions without being exposed to the cold white LED (no LID group). Retinal functionality was assessed by electroretinography (ERG) eight days after LID. Animals were then sacrificed for histopathological evaluations and quantification of human TF in ocular fluids.

### 2.6. N-Methyl-N-Nitrosourea (MNU)-Induced Retinal Degeneration Model in Rat

N-methyl-N-nitrosourea-induced retinopathy was induced as previously reported [39]. Briefly, eight-week-old male Sprague Dawley rats received a single intraperitoneal injection (i.p.) of N-methyl-N-nitrosourea (MNU) dissolved in 0.9% NaCl (Sigma-Aldrich, St. Louis, MI, USA) at the dose of 60 mg/kg body weight, while age-matched control animals received a single i.p. injection of vehicle (no MNU). Animals were sacrificed 3 or 7 days following the injections for histopathological evaluation of the retina (*n* = up to 5 rats/group/timepoint).

### 2.7. Electroretinogram (ERG) Recordings

Animals were dark-adapted for at least 15 h prior to ERG and all manipulations were performed under dim red light. Rats were anesthetized by intramuscular injection of a mixture of ketamine and xylazine. Pupils were dilated with topical 0.5% tropicamide. ERGs were recorded simultaneously in both eyes using a rodent full-integrated ERG system (Celeris, Diagnosys, Cambridge, UK). Saline lubricant eye drops were added to the end of the electrode and on the cornea to ensure optimal contact between the eye and the electrode. Electrode stimulators were placed directly onto the surface of the eye from the front and down, ensuring the whole eye was covered and there was good contact to the eye. For scotopic ERG in the dark-adapted state, flash intensities ranged from 0.01 to 3 cd·s/m^2^. For photopic ERG in the light-adapted state, flash intensities ranged from 3 to 10 cd·s/m^2^. Amplitudes of a-waves (negative waves) were measured from the baseline to the bottom of the a-wave trough, and b-wave amplitudes (positive waves) were measured from the bottom of the a-wave trough to the peak of the b-wave. Implicit times of the a-and b-waves were measured from the time of stimulus to peaks. Results were expressed in microvolts (µV) for amplitudes and milliseconds (ms) for implicit times. The values obtained for all eyes from the same experimental group were average and values are expressed as mean ± sem.

### 2.8. Histology

Oriented ocular globes were fixed for at least 24 h in Hartman’s fixative solution (Sigma-Aldrich, St. Louis, MI, USA) and embedded in paraffin. Five-µm sagittal sections containing optic nerve (ON) were performed and stained with hematoxylin and eosin (HE). HE sections from 3 consecutive slides were examined by light microscopy at a magnification of 20× and color images were obtained using a NanoZoomer-XR Digital slide scanner (Hamamatsu Photonics, Hamamatsu city, Japan). Images were analyzed using NDP View software. The outer nuclear layer (ONL thickness) in the LID model or the total retinal thickness (from the internal limiting membrane to the retinal pigment epithelium) and the outer retinal thickness (from the outer plexiform layer to the retinal pigment epithelium) in the MNU rat model were measured every 500 µm from the ON to the inferior and the superior ciliary processes. Thickness profiles along the retina were generated by averaging, for each distance, the values obtained for all eyes, as previously described [30] and values are expressed as mean ± sem. The photoreceptor ratio was calculated as the percentage of outer retinal thickness/total retinal thickness, as previously described [40].

### 2.9. Immunochemistry and TUNEL Assay

Immunofluorescent staining was performed on 5-µm-thick paraffin sections. Dewaxed slides were incubated with different primary antibodies: mouse anti-Glial fibrillary acid protein (GFAP, Sigma-Aldrich, St. Louis, MI, USA), rabbit anti-anti-ionized calcium-binding adapter molecule 1 (Iba1, Wako, Richmond, VA, USA), mouse anti-CD68 (Bio-Rad, Hercules, CA, USA), rabbit anti-cone arrestin (Sigma-Aldrich, St. Louis, MI, USA), and anti-serum albumin (MyBiosource, San Diego, CA, USA). Control sections were incubated without primary antibodies. The corresponding Alexa-conjugated secondary antibody (Thermo Fisher Scientific, Carlsbad, CA, USA) was used to reveal the primary antibodies. Final counterstaining with 4.6-diamidino-2-phenylindole (DAPI, Sigma-Aldrich, St. Louis, MI, USA) was performed to locate retinal structures. Slices were mounted with a Fluoromount mounting medium (Sigma Aldrich, St. Louis, MI, USA) under a lamella for microscopic examination using a fluorescence microscope with apotome module (Zeiss, Oberkochen, Germany), a fluorescence microscope with Spinning Disk (CSU-Xi, Intelligent Imaging Innovations, Denver, CO, USA), or a NanoZoomer-XR Digital slide scanner (Hamamatsu, Japan). Cone photoreceptors’ quantification was done by masked counting for each retinal section of the number of cone-arrestin-positive cells in the outer nuclear layer.

The TUNEL assay was performed on 5-µm-thick paraffin sections using a commercial apoptosis detection kit (Promega, Madison, WI, USA) and following the manufacturer’s instructions. Images were recorded with a NanoZoomer-XR Digital slide scanner (Hamamatsu, Japan) at 20-fold magnification and the fluorescent staining level in the whole retina was quantified using Image J software.

### 2.10. Retina/RPE/Choroid Flat Mounts

Following transversal sectioning of the globe, the anterior segments, lens, and vitreous were removed, and five radial cuts were made in the remaining eyecups containing retina, RPE, choroid, and sclera. Immunofluorescent staining was performed using a rabbit anti-zonula occludens-1 antibody (ZO-1, Thermo Fisher, Waltham, MA, USA) and corresponding Alexa-conjugated secondary antibody (Life Technologies, Carlsbad, CA, USA). Retina/RPE/choroid complexes were flat-mounted with a Fluoromount mounting medium (Sigma Aldrich, St. Louis, MI, USA) for microscopic examination using a Spinning Disk (CSU-Xi).

### 2.11. MDA Content Assay in Ocular Fluids

Quantification of malondialdehyde (MDA) in rat ocular fluids was performed using a method adapted from Giera and colleagues’ publication [41]. Diluted samples underwent alkaline hydrolysis and were then neutralized in the presence of perchloric acid. Derivatization was performed using the 2-amonoacridone, as the derivative agent, in a citrate buffer solution at 40 °C. Following cooling, the MDA level was determined by HPLC. Methyl-MDA was used as standard and the lower limit of quantification was defined as 25 nM. Values were expressed as mean ± sem.

### 2.12. Statistical Analysis

To take into account the correlation between the 2 eyes of the same rat, a mixed linear model was used to define a treatment effect on ONL thickness and ERG parameters in the LID rat model. Statistical analyses were performed using SAS 9.4 software and differences between groups and interactions were declared significant at the 5% level of significance.

For the other parameters, statistical analyses were performed using Prism 8 software (GraphPad). Tests for normality for each group were performed using the Shapiro–Wilk test. For normal distribution, one-way ANOVA followed by Tukey’s multiple comparisons test were used to define differences between groups. For non-normal distribution, the Kruskal–Wallis test followed by Dunn’s multiple comparisons test were used to define differences between groups. Differences were declared significant at a 5% level of significance.

## 3. Results

### 3.1. pEYS611 Administration Allows Sustained Intraocular Production of Human Transferrin in Rats and Rabbits

**Figure 2:**
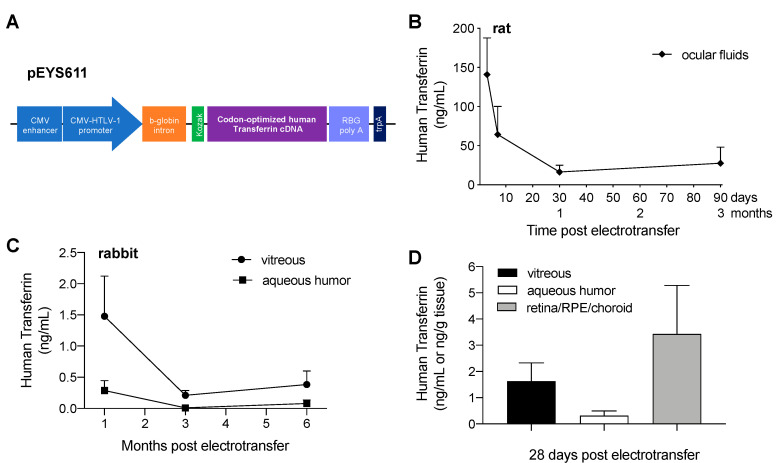
pEYS611-driven sustained human transferrin (TF) intraocular production in rats and rabbits. (**A**) The human TF expression cassette is composed of a human cytomegalovirus (CMV) enhancer, a chimeric CMV-HTLV (human T-cell leukemia virus)-I ubiquitous promoter, the ß-globin intron, and a Kozak sequence driving the expression of the human TF. (**B**,**C**) Human TF expression profiles in rat ocular fluids (**B**, *n* = 5–7 animals per timepoint) and rabbit aqueous humor and vitreous (**C**, *n* = 3–9 animals per timepoint) after bilateral ciliary muscle administration of pEYS611 at the doses of 30 and 45 µg/eye, respectively, demonstrated sustained expression of human TF for at least 3 and 6 months, in rat and rabbits, respectively. (**D**) Human TF distribution in rabbit eyes 28 days following pEYS611 administration demonstrated that the expressed protein reaches the back of the eye. Values are presented as mean ± sem.

Electrotransfection of pEYS611 plasmid (Figure 2A; 30 µg/eye) in the rat ciliary muscle allowed for the expression and secretion of human transferrin (TF) in ocular fluids (vitreous and aqueous humor), with an average maximal concentration of 141 ± 47 ng/mL reached within three days after the administration (Figure 2B). The protein was still measured 90 days following plasmid electrotransfection.

In rabbits, the maximal concentration of human TF in both vitreous and aqueous humor was reached within five days following pEYS611 administration at the dose of 45 µg/eye with the concentration being 5-fold higher in the vitreous than in the aqueous humor 28 days following the administration (Figure 2C; 1.6 ± 0.7 ng/mL in vitreous vs. 0.31 ± 0.2 ng/mL in aqueous humor). Six months after plasmid administration, human TF was still quantified in the vitreous of 75% of the treated eyes, allowing for a sustained expression of the protein, with an exposure and mean residence time of 184,061 pg.d.ml-1 and 58 days, respectively. Human TF was also quantified within two days in retina/RPE/choroid complex (data not shown), demonstrating that human TF produced and secreted by the ciliary muscle cells rapidly penetrated into the retina. A high TF protein concentration was observed in the retina/RPE/choroid complex 28 days following plasmid administration (Figure 2D; 3.4 ± 1.9 ng/g of tissue) and was still high at 6 months in this compartment (2.5 ± 1.3 ng/g of tissue vs. 0.7 ± 0.4 and 0.1 ± 0.1 ng/mL in vitreous and aqueous humor, respectively), demonstrating a high exposure (336,061 pg·d·g^−1^) and long residence time (73 days) of human TF in the posterior segment of the eye.

### 3.2. pEYS611 Preserves Photoreceptor Cells from Photo-Oxidative Damage

**Figure 3:**
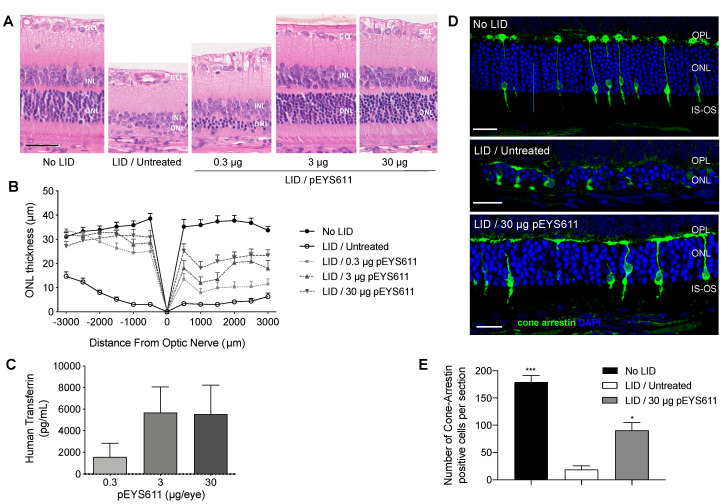
Dose-dependent effects of pEYS611 administration in the rat light-induced damage (LID) model. Rats received a single ciliary muscle electrotransfection of pEYS611 at the dose of 0.3, 3, or 30 µg/eye on day (-) 3 (D-3) or left untreated (*n* = 8 animals/group). Except for the unexposed animals (no LID), retinal degeneration was induced on D0 by 24 h of bright light exposure (6500 lux). Dark- and light-adapted electroretinography (ERG) responses were recorded simultaneously from both eyes on D8. Following ERG recordings, animals were sacrificed for analysis of human TF levels in ocular fluids and histological evaluations. (**A**) Representative images of retina section (superior pole) stained with hematoxylin eosin. Scale bar, 50 µm. (**B**) ONL thickness was measured every 500 µm from the optic nerve (0) to the inferior (−) and superior (+) poles of the retina. Compared with no LID control animals, the ONL of LID/untreated animals was thinner. Treatment with pEYS611 resulted in a dose-dependent preservation of the ONL, correlating with human TF levels measured in ocular fluids (**C**). (**D**) Representative images of cone-arrestin staining at a distance of 1500–2000 µm from the optic nerve in the superior part of the retina (4.6-diamidino-2-phenylindole (DAPI) in blue/cone arrestin in green). Scale bar, 25 µm. (**E**) The number of cone-arrestin-positive cells per retinal section was significantly higher in LID/pEYS611-treated animals compared to LID/untreated rats. All values are presented as mean ± sem. * *p* < 0.05; *** *p* < 0.001 using Dunn’s multiple comparisons test versus LID/untreated group. IS, inner segment; ONL, outer nuclear layer; OPL, outer plexiform layer; OS, outer segment.

**Figure 4:**
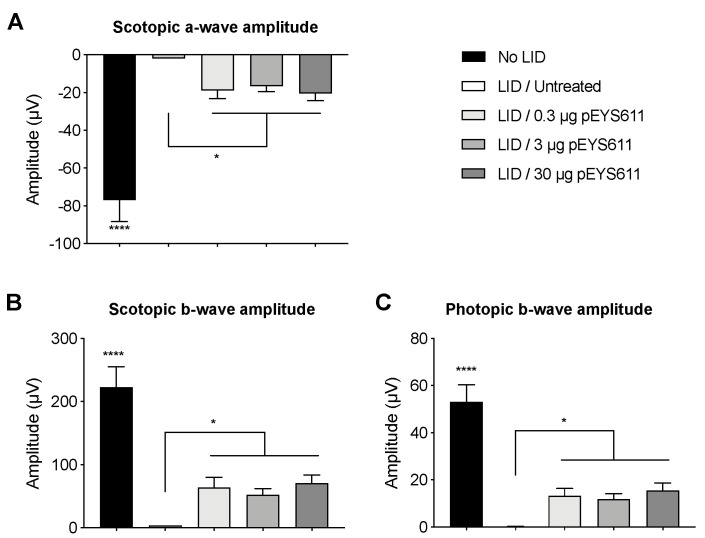
pEYS611 maintains retinal function in the rat LID model. Rats received a single ciliary muscle electrotransfection of pEYS611 at the dose of 0.3, 3, or 30 µg/eye on D-3 or left untreated (*n* = 8 animals/group). Except for unexposed animals (no LID), retinal degeneration was induced on D0 by 24 h of bright light exposure (6500 lux). Dark- and light-adapted ERG responses were recorded simultaneously from both eyes on D8. (**A**,**B**) Average amplitude (µV) of scotopic a-and b-waves from ERG responses elicited by a stimulus intensity of 3 cd·s/m^2^ and (**C**) amplitude of photopic b-wave from ERG responses elicited by a stimulus intensity of 10 cd·s/m^2^ are presented as mean ± sem. * *p* < 0.05; **** *p* < 0.0001 using a repeated measure mixed model and determination of confidence intervals versus LID/untreated group.

We previously reported the protective effect of intravitreal injection of recombinant TF in the light-induced retinal damage (LID) rat model, a well-established model of oxidative stress-induced retinal degeneration [30]. Here, we wished to confirm the protective effect of pEYS611-driven TF delivery in this model. pEYS611 was electrotransfered at doses of 0.3, 3, or 30 µg/eye in the rat ciliary muscle three days prior the LID induction performed on day 0 (D0) to achieve maximum TF expression at the time of disease initiation. ERG and ONL thickness were measured 8 days following light exposure. As expected, light exposure resulted in an extended loss of photoreceptor cells as evidenced by a reduction of 77% to 88% of the ONL thickness in the inferior and superior poles of the retina, respectively (Figure 3A,B). Notifiable changes in the morphology of cone photoreceptors were also observed following light exposure (Figure 3D). Almost all cones had no inner and outer segments, axon, and terminal pedicle. The remaining inner and outer segments were highly shortened and disorganized. Similarly, the remaining terminal pedicles were smaller and flatter, directly abutting cone cell bodies. The number of cone photoreceptors appeared dramatically reduced in response to light compared to the non-exposed control group as demonstrated with the cone arrestin-positive cells counting (18.9 ± 7.0 vs. 179.5 ± 11.8 cone arrestin-positive cells per whole retinal section, *p* = 0.0003, Figure 3E). In accordance with morphological modifications of the photoreceptor cell layer, a dramatic deterioration of scotopic and photopic b-wave amplitudes was observed when compared to unexposed control animals (Figure 4, *p* < 0.0001). These results confirmed the induction of severe retinal degeneration after light exposure in these experimental conditions.

pEYS611 treatment at the three doses tested led to the preservation of the ONL (Figure 3A). On the inferior part of the retina, the ONL thickness of all pEYS611-treated animals was significantly thicker than that of exposed untreated control animals (*p* < 0.0001), with the average ONL thickness corresponding to about 80–90% of the average ONL thickness of unexposed control animals regardless of the tested dose (Figure 3B). A significant preservation of the ONL on the superior pole of the retina was also observed, with protective effects correlating with the pEYS611 dose and TF intraocular levels (Figure 3C). Indeed, the average ONL thickness in pEYS611-treated animals at the dose of 0.3, 3, and 30 µg/eye corresponded to 30%, 49%, and 63%, respectively, of the average ONL thickness of the unexposed control animals (*p* < 0.0001). A significant increase of the cone photoreceptor density was also observed in comparison to untreated exposed animals (90.6 ± 14.3 cone arrestin-positive cells per whole section, *p* = 0.0498; Figure 3E). Despite the shorter axon length and flatter terminal pedicle, the surviving cone photoreceptors displayed a similar morphology to those observed in the control unexposed animals, with preserved cell body sizes and preserved inner and outer segment morphologies (Figure 3D). pEYS611 treatments also led to the preservation of both the mixed rod-cone and cone-mediated ERG responses, confirming a functional beneficial effect in treated animals (Figure 4). In scotopic conditions, a- and b-wave amplitudes were significantly increased in all pEYS611-treated animals in comparison to untreated LID animals (*p* < 0.05). Thus, pEYS611 administration significantly rescued from 24% to 26% of the photoreceptor response and from 23% to 32% of the internal retinal response of a normal nonexposed retinal light response. Similarly, the photopic b-wave amplitude was significantly increased by pEYS611 treatments in comparison to untreated exposed animals (*p* < 0.05), with a preservation of 22% to 29% of a normal unexposed retinal light response. In both scotopic and photopic conditions, no functional dose–response was evidenced.

**Figure 5:**
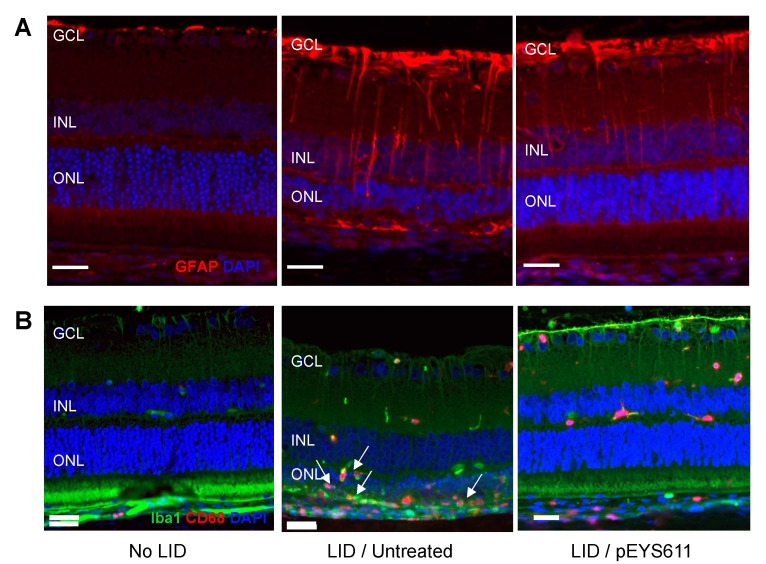
pEYS611 diminishes light-induced macroglia activation and microglia/macrophage retinal migration. Rat received a single ciliary muscle electrotransfection of pEYS611 (30 µg/eye) on D-3 or left untreated (*n* = 8 animals/group). Except for unexposed animals (no LID), retinal degeneration was induced on D0 by 24 h of bright light exposure (6500 lux). anti-Glial fibrillary acid protein (GFAP) and Iba1/CD68 staining was performed on D8. (**A**) Representative images of GFAP staining (red) in the inferior central part of the retina. (**B**) Representative images of Iba1 (green) and CD68 (red) immunostaining in the inferior central part of the retina. Arrows indicate microglial cells and macrophages accumulating in the outer retina of LID/untreated animals. Scale bar, 25 µm. GCL, ganglion cell layer; INL, inner nuclear layer; ONL, outer nuclear layer.

In response to light exposure, intense GFAP immunoreactive profiles were observed in astrocytes (located in the GCL) and in Müller cell processes extended through the inner nuclear layer (INL), demonstrating macroglia activation representative of the extent of retinal damage (Figure 5A, middle). In addition, light exposure led to microglial activation and migration in the outer retina and positive-CD68 macrophages’ infiltration in the sub-retinal space, representative of an intense inflammatory response (Figure 5B, middle). In contrast, pEYS611 treatment reduced macroglial activation, as shown by a lower GFAP staining in treated animals (Figure 5A, right). Microglial cells’ migration into the outer retina was also reduced following pEYS611 administration, and most of the IBA/CD68 labelled cells remaining properly localized in the inner retina, while infiltrated macrophages were no longer observed in the subretinal space (Figure 5B, right).

### 3.3. pEYS611 Preserves RPE Cells from Photo-Oxidative Damage

**Figure 6:**
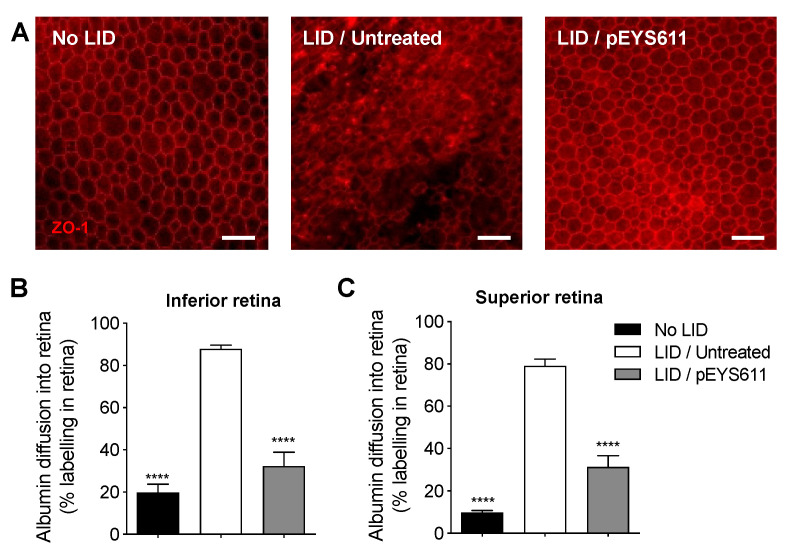
pEYS611 preserves the integrity of the outer BRB following light damage. Rat received a single ciliary muscle electrotransfection of pEYS611 (30 µg/eye) on D-3 or left untreated (*n* = 8 animals/group). Except for unexposed animals (no LID), retinal degeneration was induced on D0 by 24 h of bright light exposure (6500 lux). ZO-1 and albumin staining were performed on D8. (**A**) Representative images of flat-mounted retina/RPE/choroid ZO-1 staining at a distance of 1500–2000 µm from the ON. Scale bar, 50 µm. (**B**,**C**) Albumin into the retina was quantified in the inferior (**B**) and superior (**C**) poles of the retina. Percentage of albumin diffusion per retina section is presented as mean ± sem. **** *p* < 0.0001 using Tukey’s multiple comparisons test versus LID/untreated group.

The RPE tight junctions were examined 8 days following light exposure on flat-mounted retina/RPE/choroid complexes immunostained for zonula occludens-1 (ZO-1), a tight-junction-associated protein. In untreated LID animals (Figure 6A, middle), the integrity of RPE tight junctions was lost, with a high degree of disruption in comparison to nonexposed animals (Figure 6A, left). In contrast, pEYS611 treatment preserved the RPE border structures. Almost the totality of the middle area of the RPE monolayer showed RPE with normal morphology (Figure 6A, right). Breakdown of the retinal epithelial barrier was then evaluated by albumin diffusion into the retina 8 days following light-induced retinal degeneration. Albumin diffusion in both the inferior and superior parts of the retina was significantly increased in untreated LID animals in comparison to no LID animals (*p* < 0.0001; Figure 6B,C). This result confirmed that, in our experimental conditions, light exposure alters the outer blood retinal barrier formed by tight junctions between RPE cells and allows the passage of albumin from the choroid to the retina, as previously described [42]. pEYS611 administration reduced by about 60–70% albumin diffusion into both the inferior and superior poles of the retina compared to untreated LID animals (*p* < 0.0001; Figure 6B,C).

### 3.4. pEYS611 Provides Benefits over Antioxidant

**Figure 7:**
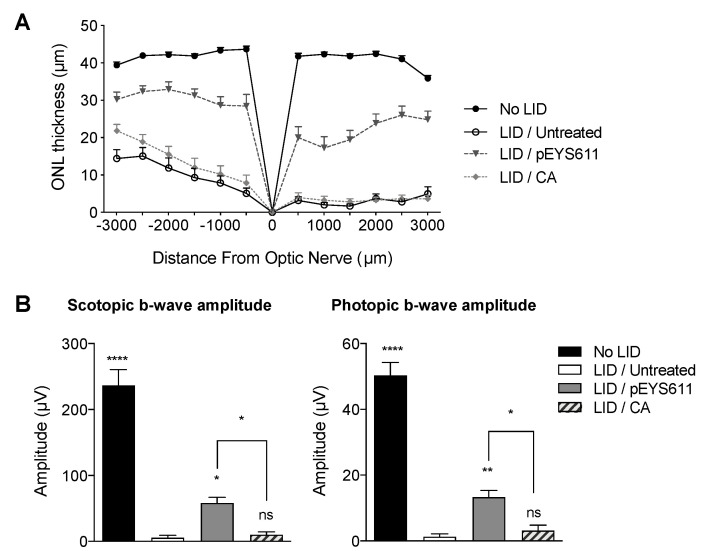
pEYS611 shows benefit over the antioxidant in the rat LID model. Rats received a single ciliary muscle electrotransfection of pEYS611 (30 µg/eye) on D-3 or daily administration of CA (25 mg/kg/day) from D-5 to D0 (*n* = 8 animals/group). Except for unexposed animals (no LID), retinal degeneration was induced on D0 by 24 h of bright light exposure (6500 lux). Dark- and light-adapted ERG responses were recorded simultaneously from both eyes on D8. Following ERG recordings, animals were sacrificed for histological examinations. (**A**) ONL thickness was measured every 500 µm from the optic nerve (0) to the inferior (−) and superior (+) poles of the retina, values are presented as mean ± sem. (**B**) Amplitude (µV) of b-waves from ERG responses elicited by a stimulus intensity of 3 cd·s/m^2^ (left) and of 10 cd·s/m^2^ (right) are presented as mean ± sem. * *p* < 0.05; ** *p* < 0.01; **** *p* < 0.0001 using repeated measure mixed model versus LID/untreated group.

The efficacy of EYS611 gene therapy was compared to the efficacy of carnosic acid (CA), an antioxidant that previously demonstrated efficacy on both retinal structure and function in the LID rat model [43]. CA (BOC Sciences, Shirley, NY, USA) was daily administered by intraperitoneal injection at the dose of 25 mg/kg/day for five days prior to light exposure as previously described [43] while pEYS611 (30 µg/eye) was electrotransfered once in the rat ciliary muscle three days prior to the light exposure. In our experimental conditions, a systemic repeated administration of CA prior to the light exposure led to significantly thicker ONL in comparison to untreated exposed animals (average ONL thickness of 9.61 ± 1.93 µm and 14.29 ± 1.89 µm, respectively; *p* = 0.0012) (Figure 7A). Nevertheless, this effect was restricted to the inferior pole of the retina. In this experiment, protective effects of pEYS611 were confirmed, with the average ONL thickness in both the inferior and superior poles of the retina being significantly higher than in CA-treated LID animals (30.66 ± 1.71 µm and 14.29 ± 1.89 µm and 21.02 ± 2.41 µm vs. 3.22 ± 0.83, respectively; *p* = 0.0002 and *p* < 0.0001), corresponding to a preservation of, respectively, 73% and 52% of the total ONL as compared to unexposed animals. In addition, about 50% of the cone photoreceptor cells were preserved in pEYS611-treated animals while preservation of cone photoreceptor cells was not observed in CA-treated animals (data not shown). In accordance with the histological results, CA treatment did not preserve ERG function while pEYS611 treatment led to a significant preservation of both scotopic and photopic b-wave amplitudes in comparison to untreated LID animals (Figure 7B).

### 3.5. pEYS611 Preserves Photoreceptors Cell in the MNU-Induced Retinal Degeneration Rat Model

To further confirm the effect of TF on oxidative stress-induced photoreceptor cell death, we tested the efficacy of pEYS611 treatment in the N-methyl-N-nitrosourea (MNU) intoxication model, an alkylating agent causing oxidative radical generation [44] and a rapid and specific apoptosis of photoreceptors in rats [45,46,47].

**Figure 8:**
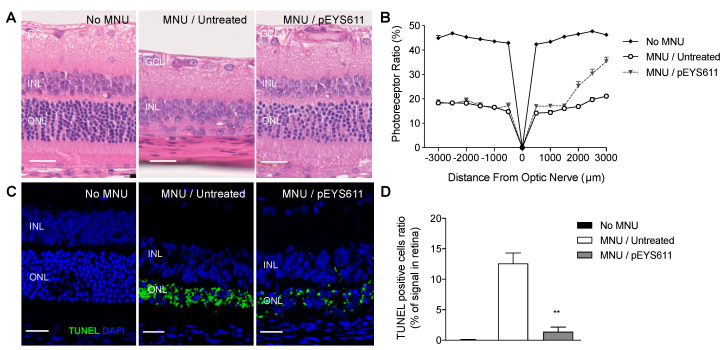
pEYS611 prevents photoreceptors from N-methyl-N-nitrosourea (MNU)-induced cell death in rats. Rats received bilateral ciliary muscle electrotransfection of pEYS611 at the dose 30 µg/eye on D-3 or left untreated (*n* = 8 animals/group). Except for not intoxicated rats (no MNU; *n* = 3 animals), retinal degeneration was induced by intraperitoneal injection of MNU (60 mg/kg) on D0. Histological analyses were performed on D3 and D8. (**A**) Representative images of the hematoxylin eosin-stained retinal sagittal section at D8. Images were performed at a distance of 2000–2500 µm from the optic nerve. (**B**) Outer retinal thickness and total retinal thickness were measured every 500 µm from the optic nerve (0) to the inferior (−) and superior (+) poles of the retina. The photoreceptor ratio was calculated as (outer retinal thickness/total retinal thickness) × 100. (**C**) Representative images of TUNEL staining on the retinal section (superior pole) performed on D3. (**D**) TUNEL immunostaining is presented as a percentage of the stained surface area over the entire retina. All values are presented as mean ± sem. ** *p* < 0.01 using Dunn’s multiple comparisons test versus MNU/untreated group. Scale bar, 25 µm. INL, inner nuclear layer; GCL, ganglion cell layer; ONL: outer nuclear layer.

One week after MNU injection, retinas from untreated rats showed thinning with selective loss of the photoreceptor cells as demonstrated by the noticeable reduction of the photoreceptor ratio in comparison to non-intoxicated control animals (average photoreceptor ration 17.9 ± 0.6% vs. 44.9 ± 0.4%, respectively; Figure 8A,B). In contrast, pEYS611 (30 µg/eye) gene delivery 3 days prior to the MNU intoxication significantly inhibited MNU-induced ONL thinning and loss of photoreceptors, especially in the mid-peripheral superior retina (31.8 ± 2.0% vs. 20.5 ± 1.1%, respectively; Figure 8B). TUNEL staining indicated the presence of 15% apoptotic cells in the whole retina in MNU untreated rats compared to nonexposed animals. pEYS611 administration reduced by about 90% the TUNEL staining in the outer nuclear layer (Figure 8C,D), demonstrating that TF could protect photoreceptors from different types of oxidative stress.

### 3.6. pEYS611 Slows down Retinal Degeneration in the RCS Rat

Finally, the efficacy of EYS611 gene therapy was evaluated on the Royal College of Surgeons (RCS) rat, a well-known model of inherited retinitis pigmentosa. In this model, mutation in the receptor tyrosine kinase gene (Mertk-/-) causes a defect in photoreceptor outer segment phagocytosis by RPE cells. The resulting accumulation of debris in the subretinal space leads to a progressive loss of photoreceptor, evidenced as early as postnatal day 18 (PND18) onward, with complete degeneration within 2 to 3 months [48,49,50].

**Figure 9:**
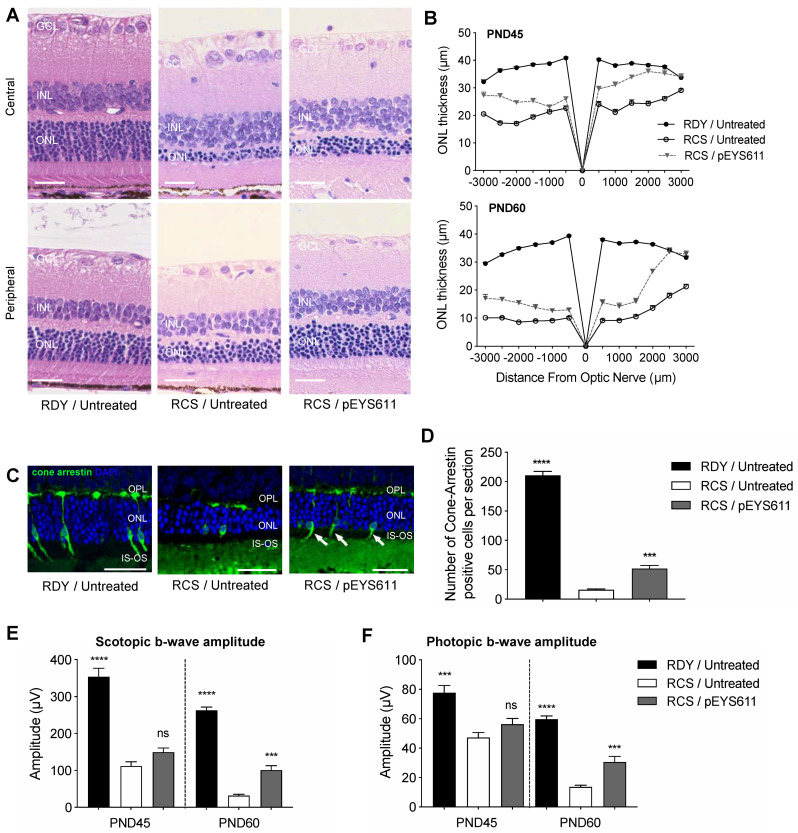
pEYS611 preserves retinal structure and function in RCS rats. At PND18, RCS rat received a bilateral injection and electrotransfection of pEYS611 in the ciliary muscle at the dose 25 µg/eye. Non-dystrophic RDY rats were used as the control and were left untreated. On PND45 and PND60, animals were sacrificed for histological examinations. (**A**) Representative images of hematoxylin/eosin-stained central (top) and peripheral (bottom) superior retina at PND60. (**B**) ONL thickness was measured every 500 µm from the optic nerve (0) to the inferior (−) and superior (+) poles of the retina. (**C**) Representative images of cone-arrestin staining in peripheral superior poles of the retina at PND60 (cone arrestin in green/DAPI in blue). (**D**) Number of cone-arrestin-positive cells per section in the whole retina was drastically reduced in RCS/untreated rats compared to RDY normal rats at PND60. pEYS611 treatment significantly preserved cones. *** *p* < 0.001; **** *p* < 0.0001 using Tukey’s multiple comparisons test versus RCS/untreated group. (**E**,**F**) Light- and dark-adapted ERG responses were recorded simultaneously from both eyes at PND45 and PND60. Amplitude (µV) of scotopic and photopic b-waves from ERG responses elicited by a stimulus intensity of 3 cd·s/m^2^ (**E**) and 10 cd·s/m^2^ (**F**), respectively, are presented. *** *p* < 0.001; **** *p* < 0.0001 using the repeated measure mixed model versus RCS/untreated group. All values are presented as mean ± sem. Scale bar, 25 µm. GCL, ganglion cell layer; INL, inner nuclear layer; IS: inner segment, ONL: outer nuclear layer, OPL: outer plexiform layer, OS: outer segment.

pEYS611 at the dose of 25 µg/g per eye was administered to RCS rats at PND18 and its effects monitored at PND45 and PND60 in comparison to untreated RCS rats and to untreated non-dystrophic age-matched RDY control rats. As expected, an extended loss of photoreceptor cells was observed in untreated RCS animals at PND45 and PND60 as evidenced by a significant reduction of the ONL thickness as compared to RDY control animals (Figure 9A,B). At PND45, the average ONL thickness was reduced in both inferior (−47%, *p* < 0.0001) and superior (−34%, *p* < 0.0001) parts of the retina in comparison to age-matched RDY rats. At PND60, ONL degeneration progressed, with a reduction of about 70% of the average ONL thickness (*p* < 0.0001, Figure 9A,B). In contrast, pEYS611 significantly slowed the progression of the disease, compared to untreated RCS rats (Figure 9A,B). As early as 27 days post-administration (PND45), the average ONL was significantly thicker in pEYS611-treated animals than in the untreated RCS animals, both in the inferior (+30%, *p* < 0.0001) and in the superior retina (+33.7%, *p* < 0.0001). Despite the progression of the disease, the ONL thickness remained significantly thicker at PND60 in pEYS611-treated RCS animals in comparison to untreated RCS rats (*p* < 0.0001).

Cone arrestin staining and positive cells quantification were performed at PND60, i.e., 42 days following pEYS611 ciliary muscle administration. Noticeable changes in the morphology of the remaining cone photoreceptors were observed in untreated RCS rats (Figure 9C). Most of the outer segments (OSs) were lost and the remaining inner segments (ISs) were swollen. Similarly, the remaining terminal pedicles were smaller and flatter, with most of them directly abutting cone cell bodies, especially in the central retina (data not shown). In addition, the total number of cone photoreceptors appeared dramatically reduced compared to non-dystrophic RDY animals (16.1 ± 1.1 vs. 210.6 ± 6.9 cone arrestin-positive cells per retinal section, *p* < 0.0001, Figure 9D). Morphological ONL preservation in the inferior and superior parts of the retina was associated with significant preservation of cone photoreceptor cells in pEYS611-treated animals (Figure 9C, right panel), with more than 3 times as many cells than in the untreated RCS group (52 1 ± 5.1 vs. 16.1 ± 1.1 cone arrestin-positive cells per retinal section, *p* = 0.0002, Figure 9D). In addition, preservation of the cell body was associated with the preservation of axons (despite shorter length) and outer segments (especially in the peripheral retina).

The extended loss of photoreceptor cells in untreated RCS animals was related to a significant progressive decrease of a-wave (data not shown) and b-wave amplitudes (Figure 9E,F) from both dark- and light-adapted ERG as compared to non-dystrophic age-matched RDY rats (*p* < 0.0001). Scotopic and photopic implicit times (regardless of the waves) were also increased by disease progression (data not shown), confirming the alteration of rod- and cone-mediated retinal function in RCS rats. pEYS611 treatment led to the preservation of both the mixed rod-cone response and cone-mediated response as demonstrated by significant higher scotopic and photopic ERG b-wave amplitudes at PND60 in comparison to untreated RCS animals (*p* < 0.001).

Finally, we looked for surrogate markers of pEYS611 biological activity in ocular fluids of RCS rats and measured the content in malondialdehyde (MDA), a common marker of lipid peroxidation that accumulates in many pathological conditions [51,52]. Ocular fluids from untreated RCS rats had a significant elevation in the mean MDA content compared to ocular fluids from RDY control animals at PND45 (62.7 ± 8.3 µM vs. 34.0 ± 3.4 µM, *p* = 0.0093). Interestingly pEYS611-treated RCS rats demonstrated a reduction in MDA content in ocular fluids when compared to untreated RCS rats (40.7 ± 11.3 µM), corresponding to a 35% reduction.

## 4. Discussion and Conclusions

Non-viral gene delivery of TF using ciliary muscle electrotransfection allows for the sustained intraocular production of human TF in both rats and rabbits. Though the produced protein is not 100% homologous to the animal species (73% and 79% for rat and rabbit TF, respectively), no sign of immune response or inflammation was observed, confirming the good safety profile of human TF as previously reported after repeated IVT injections in rat [30]. Not only human TF was measured in the vitreous for at least 3 and 6 months in rats and rabbits after one single electrotransfection, but pEYS611 showed protective effects in the long term in the RCS rat (up to 42 days after electrotransfection), demonstrating that human TF remains biologically active. In addition, the expressed TF protein widely distributed in all compartments of the eye and concentrated in the outer retina, allowing significant preservation of the retina in different models of retinal degeneration. Similar observations have been made following ciliary muscle electrotransfection of plasmids coding for other proteins, such as anti-TNF in animal models of uveitis [34,53], anti-VEGF in a rat model of choroidal neovascularization [54], and neurotrophic factors in rat models of retinal dystrophies [55], confirming that plasmid electrotransfection into the ciliary muscle is a valuable approach to deliver any therapeutic proteins to the retina.

To gain confidence in the therapeutic potential of TF non-viral gene therapy for the treatment of retinal degeneration and because no animal models fully replicate human conditions, the effect of pEYS611 treatment has been evaluated in several induced and inherited models of retinal degeneration. The light damage in rats is a well-established model of retinal degeneration inducing photoreceptor-specific cell death, breakdown of the blood–retina barrier, oxidative stress, inflammation, and subsequent loss of functional vision [56,57], recapitulating some of the hallmarks of dry AMD and RP [58,59,60]. We and others also reported modification of iron metabolism and iron overload in the injured outer retina following light damage [29,30,61], making this model a relevant model to evaluate the pharmacodynamics effects of pEYS611. Indeed, we previously showed that intravitreal injection of a high dose of human TF was efficient to rescue photoreceptors from light-induced cell death, with morphological and functional preservation being associated with reduced iron overload and decreased oxidative stress-induced heme oxygenase expression [30]. Here, TF non-viral gene therapy similarly and very consistently protected the inferior outer retina from LID even when delivered at a very low dose while higher TF protein concentrations were required to fully preserve the superior retina. This regional effect of pEYS611 is in agreement with a previous observation reporting higher susceptibility of the superior retina to light damage [62]. Increasing the plasmid dose from 0.3 to 3 or 30 µg/eye induced an increase in human TF vitreous levels, with no statistically significant difference between 3 or 30 µg, suggesting that in the rat eye, other strategies could be used to reach higher protein levels, such as repeated administrations in other muscle quadrants [33,36]. Although a clear morphologic dose–response effect was observed between 0.3 and 3 µg of pEYS611, with a higher number of cones protected, no significant change was noted with increasing the dose from 3 to 30 µg/eye, which correlates with TF levels. Interestingly, at a functional level, a similar improvement in the retinal light response was measured with either the lowest or the highest dose of plasmid, suggesting that even a very low dose of TF might be sufficient to neutralize iron excess and subsequent cell death. Although cones were better preserved with the high dose of pEYS611, some cones still presented morphological abnormalities and might be not functional, possibly explaining the apparent discrepancy between the morphological and functional results. Interestingly, at the highest dose of plasmid used, TF not only protected photoreceptors from cell death but also prevented the RPE barrier breakdown, as witnessed by reduced albumin leakage, preserved RPE structure, and reduced retinal inflammation. The anti-inflammatory properties of TF have already been shown in the ischemic brain [63], but the protective role of TF on the RPE barrier had not been evidenced previously, confirming that TF might have additional non-iron-mediated effects as shown by transcriptomic analysis of TF effects in a retinal detachment model [6]. The superiority of TF over CA, a well-known antioxidant, is in line with this observation. In our hands, the protection achieved with CA was less prominent compared to previously published results [43]. This discrepancy might be explained by the more drastic light damage conditions used in our study, i.e., 24-h exposure at 6500 lux versus 5-h exposure at 5000 lux in Rezaie and colleagues’ study [43]. In line with the neuroprotective properties of TF, pEYS611 at 30µg/eye significantly reduced photoreceptor apoptosis induced by MNU with an efficacy comparable to that reported following administration of caspase 3 or calpain inhibitors [40,64]. Here again, a regional protective effect of pEYS611 was observed in line with the less pronounced toxicity of MNU in both the peripheral and superior retina [39]. Taken together, our results and others support broader neuroprotective functions of TF beyond its iron chelation activity.

These results are of great therapeutic importance for the treatment of retinal degenerative disorders with still unmet needs. Beyond gene mutation, accumulating evidence suggests that oxidative stress is a key contributor to cone degeneration in RP [65,66,67]. Gene replacement using viral gene therapy is only possible for a very limited number of genotyped RP patients, leaving most patients without any therapeutic options. Here, we first report the benefit of TF in the RCS rat model of RP, which presents spontaneous retinal degeneration due to a mutation in the Mertk gene [49,68], and sharing many similarities with autosomal recessive RP (arRP) caused by mutations in MERTK in humans. When administered at disease onset, pEYS611 significantly delayed retinal degeneration and vision loss. Interestingly, the benefit observed following pEYS611 treatment was in the same range to that reported following subretinal injection of viral vectors aiming at correcting the Mertk gene and administered at much earlier timepoints [69,70], reinforcing the neuroprotective potential of such a non-viral gene therapy approach for RP patients. The benefit of TF is also not specific of a particular RP mutation or gene. Indeed, we previously reported the benefit of TF in rd10 mice, a murine model of inherited RP due to the mutation of the Pde6b gene [19] and in P23H rats, carrying a mutation of the Rho gene [30]. Taken together, these results strongly support further development of pEYS611 gene therapy to slow down photoreceptor degeneration and to preserve visual performances in all RP patients regardless of their underlying genetic defect. While RP is most often characterized by a slowly degenerative process complicating the clinical evaluation of a new drug candidate, we identified the aqueous humor MDA level as a potential surrogate biomarker of the activity of TF. Whether the MDA content in aqueous humor of RP patients is increased remains to be demonstrated. In a very limited cohort of RP patients, Campochiaro and colleagues did not observe any changes in the MDA content both in aqueous humor and in serum [67] while they showed a significant reduction in the reduced to oxidized glutathione (GSH/GSSG) ratio in aqueous humor and a significant increase in the aqueous protein carbonyl content. The authors suggested that most lipids are too hydrophobic to enter into the aqueous humor, but our results in RCS rats, using another method to detect MDA, suggest otherwise. Indeed, whilst most MDA assays are based on its derivatization with thiobarbituric (TBA) [71], a more accurate and sensitive method based on selective derivatization with 2-aminoacridone (2-AA) combined with HPLC detection was used herein (Daruich et al., 2020, Molecular Vision In press). This method, initially optimized for the measurement of free and protein-bound MDA in human tears, might be a valuable tool to (re)investigate the MDA content in aqueous humor and/or vitreous of RP patients.

Beyond RP, pEYS611 gene therapy approach targeting iron-mediated ocular oxidative stress has a strong upside potential for the treatment of more frequent invalidating retinal degenerative diseases, such as dry AMD, a leading cause of blindness for which there is still no treatment option. The causal link between AMD pathogenicity and iron overload remains to be established, but some evidence is provided supporting that iron homeostasis dysregulation is associated with AMD [72]. Retinal iron levels increase with age [73], AMD-affected maculae have increased iron especially in the RPE and Bruch’s membrane compared to healthy age-matched controls [10,12], and iron levels in aqueous humor were increased by more than two-fold in patients with dry AMD [13]. An upregulation by about 2-fold of both TF transcript and protein is also described in AMD patients [14]. Melanin usually buffers the iron content and preserves the RPE and the choroid from a pro-oxidant environment. However, its content in RPE decreases with age while iron accumulates in melanosomes, leading to the formation of free radicals in aging retinas [74]. In addition to the obvious potential of iron chelation to protect photoreceptors cells, prevention of the deleterious effects of iron-induced damages on the blood–retina barrier is another interest of TF non-viral gene therapy to treat AMD since the degeneration of the RPE cells is closely related to the pathogenesis of non-exudative AMD. Aforementioned, pEYS611 administration preserves the integrity of the outer blood retinal barrier formed by tight junctions between RPE cells, reducing the diffusion of albumin from the choroid to the retina.

In conclusion, pEYS611 appears as a promising approach for the treatment of retinal degeneration. By combining a non-viral gene therapy and a safe delivery approach, electrotransfection of plasmids into the ciliary muscle provides huge advantages over current viral vectors in development for RP or other retinal degeneration: (i) The administration procedure is minimally invasive and does not require subretinal surgery, thus allowing intervention at an earlier stage of the disease with no risk of retinal injury; (ii) the lack of immunogenicity of plasmid DNA allows for re-dosing; and (iii) this delivery approach allows for a fine-tuning of the expressed therapeutic protein by modulating the plasmid dose and/or the surface of transfected muscle [36], offering the possibility to adapt the treatment to the patient’s disease progression. Finally, plasmid DNA have no limited cargo capacity, offering the possibility to deliver almost any therapeutic proteins or even to combine therapeutic proteins on the same plasmid to address multiple pathogenic pathways.
